# A Finite Element Method Study of Stress Distribution in Dental Hard Tissues: Impact of Access Cavity Design and Restoration Material

**DOI:** 10.3390/bioengineering11090878

**Published:** 2024-08-29

**Authors:** Mihaela-Roxana Boțilă, Dragos Laurențiu Popa, Răzvan Mercuț, Monica Mihaela Iacov-Crăițoiu, Monica Scrieciu, Sanda Mihaela Popescu, Veronica Mercuț

**Affiliations:** 1 Department of Prosthetic Dentistry, University of Medicine and Pharmacy of Craiova, 200349 Craiova, Romania; mihaelabotila09@yahoo.com (M.-R.B.); monica.craitoiu@umfcv.ro (M.M.I.-C.); monica.scrieciu@umfcv.ro (M.S.); veronica.mercut@umfcv.ro (V.M.); 2 Department of Automotive, Transportation and Industrial Engineering, Faculty of Mechanics, University of Craiova, 200478 Craiova, Romania; 3 Department of Plastic Surgery, University of Medicine and Pharmacy of Craiova, 200349 Craiova, Romania; 4 Department of Oral Rehabilitation, University of Medicine and Pharmacy of Craiova, 200349 Craiova, Romania; sanda.popescu@umfcv.ro

**Keywords:** access cavity, endodontic treatment, finite element analysis, stress analysis

## Abstract

The design of the access cavity is an important factor in endodontic treatment for the further evolution of the tooth. The objective of this study was to highlight the most favorable access cavity design (TrussAC, UltraAC, TradAC, CariesAC, ConsAC, RestoAC) based on the stress distribution on virtual models of mandibular molars. To achieve the objectives of the study, four series of virtual models of six molars were made. The first two series of external virtual models were obtained based on the three-dimensional scanning of the molars before the access cavity preparation and after their restoration, to obtain the density of the restorative materials. Internal morphology was added to the next two series of virtual models and after that, materials were added, specific for root canal obturation and coronal restoration. The simulations were performed for two coronary restoration materials, bulk fill composite and amalgam. The results showed, based on the stress maps, that the highest values were recorded for CariesAC and the lowest values for UltraAC. Comparing the two restorative materials, the lowest level of stress, strains, and displacements was highlighted in the case of UltraAC, TradAC, and ConsAC cavities for amalgam. The results obtained in this study should guide doctors towards a conservative attitude with the preservation of as much hard tissue as possible and the differentiated use of restorative materials according to the amount of tissue lost when preparing the access cavity.

## 1. Introduction

Creating the access cavity is the first “invasive” step of the endodontic treatment with a determining role in the subsequent stages [1] and in the endodontic treatment result [2], playing an important role in the longevity of the treated tooth. The American Association of Endodontics (AAE) defined the access cavity in 2020 as “the preparation of the tooth to gain access to the root canals for the purpose of cleaning, preparation and obturation” [3].

Traditional access cavities, due to their size, focused primarily on the needs of operators to identify/locate root canals and produce better disinfection. However, the size of these traditional cavities does not allow the preservation of the dental structure and reduces the fracture resistance of teeth [4]. Traditional access cavities involve complete removal of the pulp chamber roof and shortening of the walls that could compromise access and visualization of the root canals during treatment [5]. Other authors [6,7] stated that this excessive removal of tooth structure is closely related to coronal fractures of teeth subjected to occlusal forces.

In recent years, the conventional endodontic approach has been challenged by minimally invasive endodontics [8]. Modern access cavity designs have recently been used to reduce the loss of hard tooth structure [9]. Minimally invasive access cavities aim to preserve the hard structure of teeth by preserving as much dentin as possible, including the roof of the pulp chamber, to prevent fracture during and after endodontic treatment [2]. This has become possible thanks to technological developments such as magnification, nickel-titanium instruments and the input of CBCT images. Thus, the minimally invasive preparation of the access cavity has become more feasible, making it possible to preserve a greater amount of dental tissue while maintaining the quality of the endodontic treatment [5].

However, Shabbir stated in 2021 that there is a lack of evidence that minimally invasive access cavity designs will improve the fracture resistance of endodontically treated teeth [10]. Also, a minimally invasive design of the access cavity can create problems in the subsequent stages of endodontic treatment, such as a reduced visibility of the pulp chamber and root canals, and low effectiveness in instrumentation and canal disinfection [2,3].

Hard tissue removal caused by creating the access cavity raises the issue of its restoration after the endodontic treatment is completed, being another factor that contributes to the resistance over time [11]. Restoring endodontically treated teeth has been and still is a challenge for dentists.

As for direct restorative materials, amalgam has been used successfully for almost 200 years due to its low cost, low wear rate, high compressive strength and long-term survival [12,13,14]. Lately, it has lost its popularity amongst some clinicians and especially amongst patients because of its toxic effect but also because of its aesthetic deficiencies [14].

Although the use of amalgam in dentistry has gradually decreased, dental faculties in Canada [15], the United States [16] and Europe [17] continue to recommend amalgam as a material for coronary restorations.

The recommendation to minimize the use of amalgam by using alternatives whenever possible has led to considerable changes in operative dentistry in recent decades, with a shift towards greater use of adhesive materials [2,18,19].

In the last few years, with advances in adhesive dentistry and the introduction of modern adhesive systems, the clinical recommendation of using a full-coverage restoration after endodontic treatment has been questioned, with the use of bulk fill composite materials increasingly being considered, or composite materials reinforced with fiberglass [20,21].

Analyzing the distribution of occlusal forces on coronary restorations, Larson found in 2014 that the mandibular molars fracture most frequently, especially the lingual cusps, due to the high concentration of forces in this region (55% of the total occlusal forces at the level of the mandibular second molar) [22].

The aim of this study was to obtain the virtual models of mandibular molars with various designs of access cavities, restored with either amalgam or composite, to simulate and compare the stress distribution in the hard tissues of the respective teeth, using the three-dimensional method with finite elements (FEM).

The present study is an “in silico” study and is based on “in vivo” models of molars. An “in silico” study is one performed on a computer or via computer simulation software [23].

The null hypothesis is that the von Mises stress distribution is not influenced by the access cavity’s design.

## 2. Materials and Methods

### 2.1. Material

#### 2.1.1. Selection and Preparation of Teeth

The present study started from six recently extracted mandibular molars, in which access cavities were prepared according to Silva’s classification [3], teeth that were later restored. The teeth included in this study were atraumatically extracted and then disinfected in 10% peroxide solution for 10 min. The teeth were cleaned by ultrasonic scaling, a professional brushing was performed, and they were rinsed with water. The teeth were kept in NaCl 0.9% until the access cavities were made [24].

The criteria for teeth selection were the following: (1) patient’s consent to participate in the study, (2) teeth extracted because of severe periodontal damage (teeth were extracted atraumatically, without damaging the hard tissues).

The study was approved by the Ethics Committee of the University of Medicine and Pharmacy Craiova. The patients gave their consent to participate in the study by signing the informed consent (No. 212/10.11.2022).

To achieve the objectives of the study, four series of virtual models of the six molars were made.

Thus, two series of external virtual models were obtained based on two three-dimensional scanning operations, before the access cavities preparation and after their restoration. These operations were necessary to obtain the external virtual models of the molars and to determine, based on the virtual models, the volume of material used to restore each type of cavity.

To determine the stress in the dental tissues of teeth with endodontic treatment and coronal restoration, starting from the previous virtual models, two more series of virtual models were created, in which the internal morphology was added and then materials specific to the root canal obturation and coronary restorations. Bruxism-specific loads were simulated for this last category of models.

**Figure 1:**
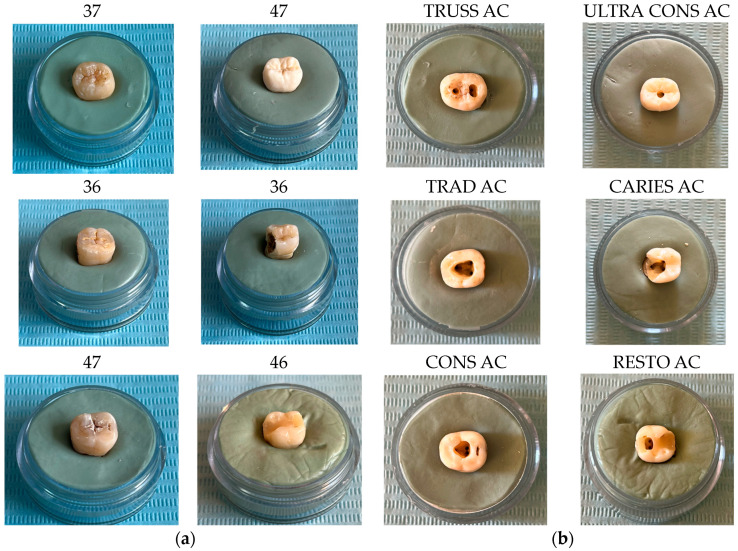
(**a**) Teeth before cavity preparation; (**b**) teeth with prepared access cavities.

The access cavity designs were made according to the particularities of the teeth (Figure 1), as follows [3]:

- Molar 37 (without carious lesions)—TrussAC;
- Molar 47 (without carious lesions)—UltraAC;
- Molar 36 (tooth with wear on the occlusal surface)—TradAC;
- Molar 36 (occluso-distal carious process)—CariesAC;
- Molar 47 (occlusal carious process)—ConsAC;
- Molar 46 (coronary obturation)—RestoAC.

The access cavities were made using the following burs: turbine round diamond bur, extra hard round bur for opening the pulp chamber, Endo-Z bur for removing the pulp chamber roof, tapered burs for finishing the walls of the access cavity [5,25].

#### 2.1.2. Hardware and Appliances

Two computing systems were used to process data, models and simulations:

- several desktop computers, with 8 GB RAM memory, INTEL Core I3 processor with a frequency of 3.7 GHz;
- a laptop type computer, with 16 GB RAM memory and INTEL Core I5 processor with a frequency of 2.6 GHz.

To create the virtual models of the molars, the 3D SYSTEMS CAPTURE 3D scanner was used, coupled with a desktop computer, having the following technical characteristics (Table 1).

An ELB300 scale with the following characteristics was used to determine the density of the materials that were used for the coronal restoration of the analyzed molars:

- Scale weight: approx. 1.25 kg
- Material: Weighing plate/SUS304 stainless steel
- Accuracy: 0.01 g
- Maximum admissible mass: 300 g
- Diameter of weighing plate: φ110 mm
- Power supply: 100 V AC adapter (included) or AA alkaline dry batteries × 6 (optional) Size: 188 × 216 × 58 mm

#### 2.1.3. Software

The Microsoft Office package was used for simple calculations, graphs and comparative analysis of the obtained values.

The Geomagic for SolidWorks program (3D Systems, Rock Hill, SC, USA) was used for the three-dimensional scanning of the molars, but also for the primary processing. This program incorporates reverse engineering methods and techniques which help to obtain complete models of the molars.

SolidWorks (Dassault Systèmes, Velizy-Villacoublay, France) was used to obtain virtual solids, which were later analyzed with FEM, which is a program that uses direct engineering methods and Computer Aided Design (CAD) techniques.

To determine the mechanical behavior of the molar models, Ansys Workbench (Ansys, Inc., Canonsburg, PA, USA) was used, a program that allows the use of the FEM method.

### 2.2. Method

The following methods were used in this study:

− Methods and techniques of reverse engineering;
− Methods and principles of CAD and direct engineering;
− Techniques and methods specific to the FEM [26];
− Methods and principles of the Mechanics of Continuous Media;
− Techniques and methods specific to the Strength of Materials;
− Principles and techniques of tooth restoration after root canal treatments [27,28,29].

### 2.3. External Virtual Models of the Selected Molars

#### The Virtual Model of the Molar with TrussAC

**Figure 2:**
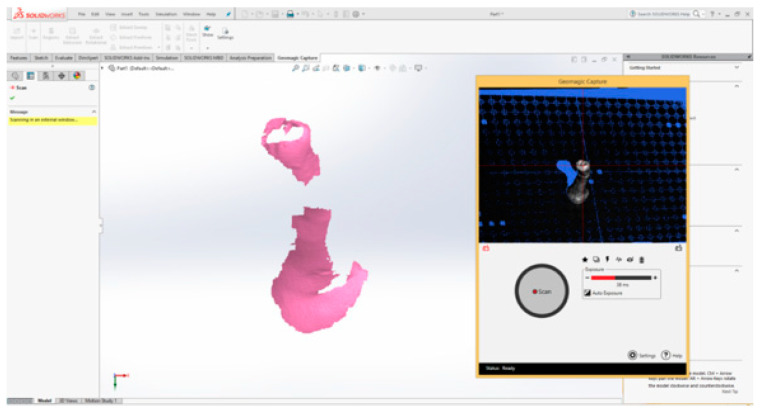
Geomagic 2019 program interface.

The 3DSystems Capture scanner was used to obtain the virtual model of the molar. Figure 2 shows the interface of the Geomagic 2019 program adapted to command the 3D scanner. The molar was fixed in Zeta Plus Putty.

**Figure 3:**
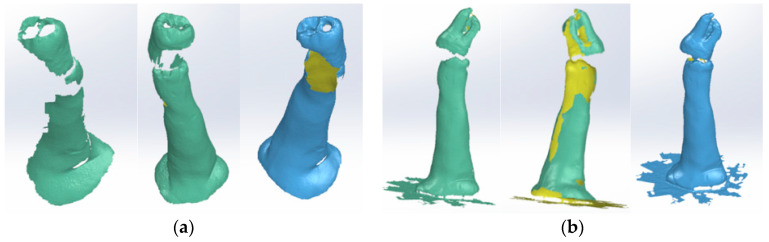
Scanning steps. (**a**) Dental crown; (**b**) roots.

Figure 3 shows some stages of the dental crown scanning operation. The molars were fixed in the holder so that the tooth roots were visible, and the scanning process continued.

**Figure 4:**
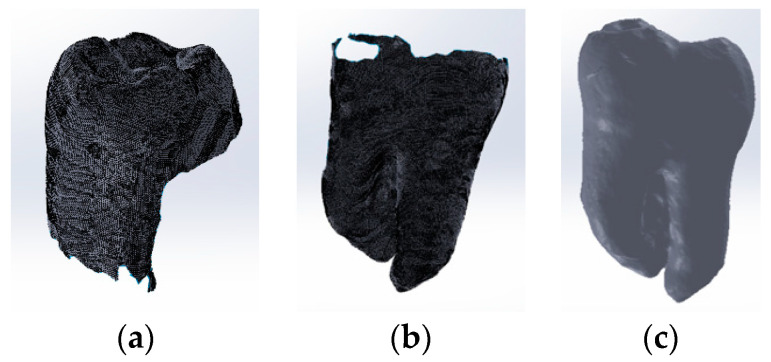
Models of: (**a**) crown; (**b**) roots; (**c**) overlaid in the Assembly module.

Next, the models were processed in Geomagic, and first, the support models were removed. The two models of the crown and the roots were loaded into SolidWorks. These patterns are shown in Figure 4a,b. The two models were loaded, one by one, into the Assembly module of the SolidWorks program. Using certain geometric elements from the two models, the model shown in Figure 4c was obtained.

**Figure 5:**
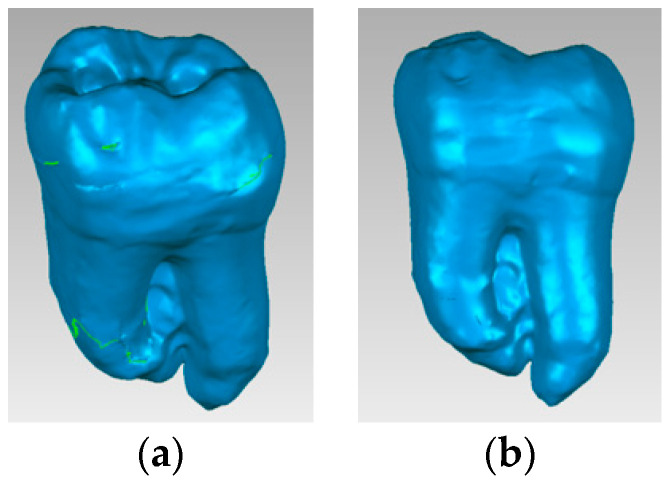
Two views of the final model of the studied molar: (**a**) lingual; (**b**) buccal.

At that time, there were two overlapping geometries on certain portions. To obtain a single surface, this model was again loaded into Geomagic, where a virtual scan was performed. In this way, only a virtual model of the molar was obtained. Finally, specific reverse engineering techniques were also used, and the result is shown in Figure 5.

**Figure 6:**
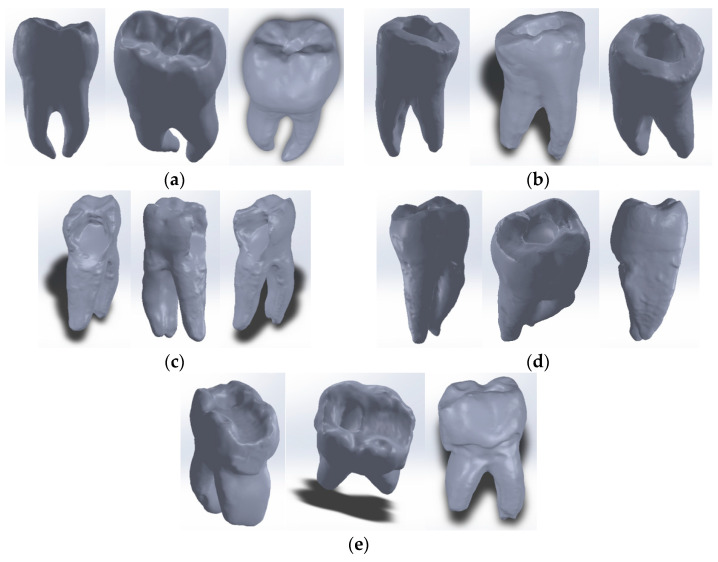
External virtual models of selected molars (three spatial views): (**a**) UltraAC; (**b**) TradAC; (**c**) CariesAC; (**d**) ConsAC; (**e**) RestoAC.

Using similar techniques and methods, characteristic specific reverse engineering [30], the external models of all molars included in this study were obtained. The images are presented in Figure 6.

### 2.4. The External Virtual Models of the Restored Molars

To obtain the external virtual models, the molars were restored using a bulk fill composite (Voco X-tra fil). After these operations of restoration, the molars were scanned three-dimensionally, again, around the dental crowns. The molars were fixed in ZetaPlus Putty.

**Figure 7:**
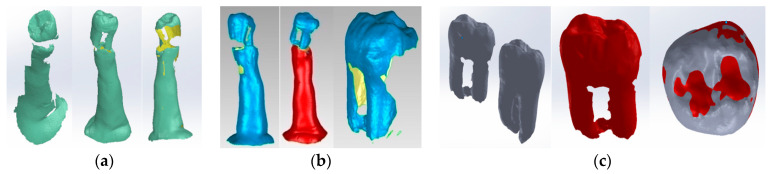
(**a**) Scanning operations applied to the crown; (**b**) virtual support removal; (**c**) superimposition of the two models (red color—surfaces that will undergo Geomagic 2019 operations).

To obtain the models of the restored molars, the scanning operations were resumed. Figure 7a shows some of these steps. In the next step, the virtual silicone supports were removed from the scanned models, as shown in Figure 7b. The models were loaded into SolidWorks, then into the Assembly module, where the models with restorations (colored in red) were overlaid on top of the models with access cavity. The steps are shown in Figure 7c.

**Figure 8:**
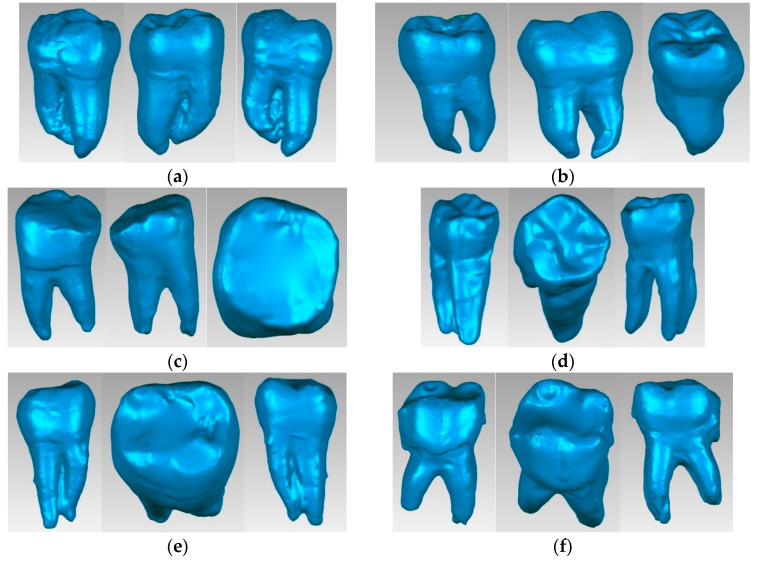
Molar models with coronal restorations made in Geomagic (three spatial views): (**a**) Molar 37; (**b**) Molar 47; (**c**) Molar 36; (**d**) Molar 36; (**e**) Molar 47; (**f**) Molar 46.

The model was imported into Geomagic, where it was virtually scanned to obtain a single model of the restored molar, as shown in Figure 8. Using similar reverse engineering techniques and methods, the external models of all restored teeth were obtained.

### 2.5. Virtual Models of Molars with Internal Anatomy

Starting from the observation that the dentin model has approximately the same geometric shapes as the enamel, but on a smaller scale, the internal anatomy of the studied molars was created. The same can be said about the dental pulp. In this sense, the Geomagic program allows the definition of those “offset” type surfaces, which can be defined relative to the entire model or to selected surfaces.

**Figure 9:**
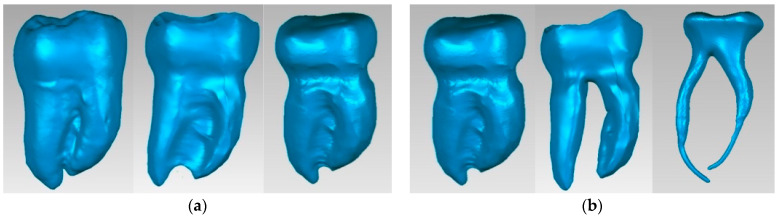
(**a**) Transformation of tooth enamel model into dentin model; (**b**) transformation of the dentin model into the dental pulp model.

To define the dentin model, the external enamel model of the intact tooth (similar to the one with restoration) was used. Thus, Figure 9a shows the stages of transformation of the enamel model into the dentin model, using mainly “offset” techniques. Similarly, the dentin model was transformed into the dental pulp model, also using addition techniques and virtual solid removal. Figure 9b shows some of the stages of dental pulp model generation.

**Figure 10:**
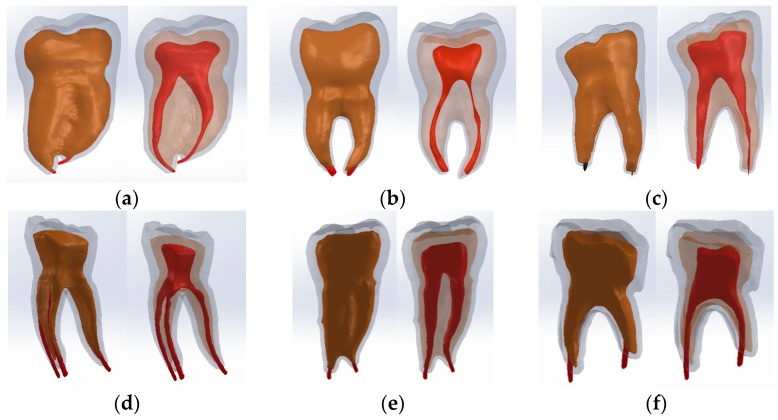
Molar models with internal anatomy (two views with different degrees of transparency): (**a**) Molar 37; (**b**) Molar 47; (**c**) Molar 36; (**d**) Molar 36; (**e**) Molar 47; (**f**) Molar 46.

By loading the three models into the Assembly module of SolidWorks and aligning them based on the reference systems, the molar models with complete internal anatomy were obtained, as can be seen in Figure 10.

### 2.6. Virtual Models of Molars with Internal Anatomy Simulated, Root Canal Filling and Coronal Restoration

**Figure 11:**
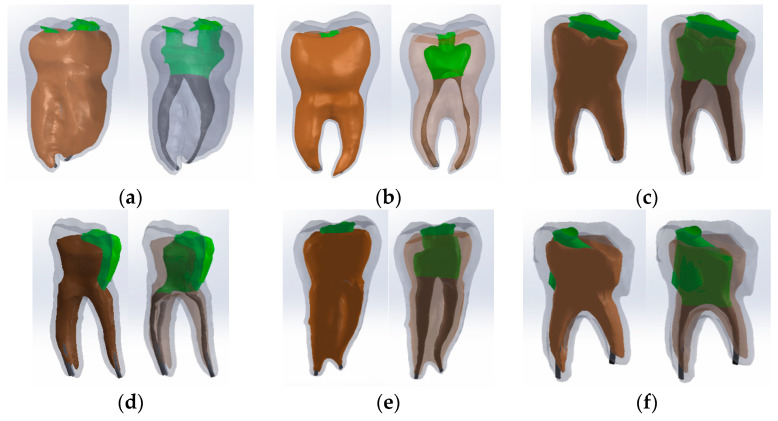
The virtual models of the molars with internal anatomy in which root canal obturation and coronal restoration were simulated (two views with different degrees of transparency and colored in green the access cavity and the pulp chamber). (**a**) Truss AC; (**b**) UltraAC; (**c**) TradAC; (**d**) CariesAC; (**e**) ConsAC; (**f**) RestoAC.

Restoration techniques were considered to obtain these models. Also, the volumes corresponding to the cavities and the upper part of the dental pulp were removed by virtual milling, using direct engineering techniques and methods. The morphology of the root pulp was used to generate the gutta-percha volume (which prefigures the root canal obturation). The so-called volume decrease (Cavity) was also used to define the specific cavities. Thus, the following virtual models were obtained (Figure 11).

### 2.7. Simulation of the Mechanical Behavior of the Analyzed Molars

#### 2.7.1. Establishing the Physico-Mechanical Properties of Materials Used in Finite Element Simulation

To obtain correct simulations, the physical and mechanical properties of the materials used in the analyses are important. These values are stored in the Engineering Data module of the Ansys Workbench program and were obtained from the selective bibliography [31,32,33,34,35,36]. Since the values for the densities of the two coronary restoration materials (composite and amalgam) were not found in the literature, it was decided to determine them experimentally.

#### 2.7.2. Determination of Densities for the Two Restorative Materials, Bulk Fill Composite Resin and Amalgam

The following mathematical relationship exists:

(1) m=ρ·V

where:

m—is the mass of the sample, measured in kg;

ρ—material density, measured in kg/$m^{3}$;

V—the volume of the material sample, measured in $m^{3}$.

The relation (1) can be expressed as follows:

(2) $$\rho =\frac{m}{V}$$

So, if the volume and mass of a sample is determined, then the density of the material is simple to determine.

For a cylindrical sample, the volume can be determined with the relation:

(3) $$V=\pi \cdot {\left(\frac{d}{2}\right)}^{2\;}\cdot h$$

d—diameter of the cylindrical sample, measured in meters;

h—the length of the cylindrical sample, measured in meters.

**Figure 12:**
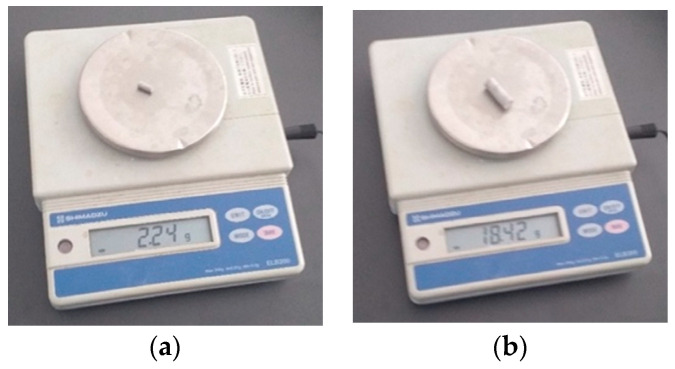
(**a**) Determination of the mass of the composite sample; (**b**) determination of the mass for the amalgam sample.

Two cylindrical samples were made, as follows:

- Composite sample with a diameter of 9.02 mm (0.00902 m), length of 33.32 mm (0.03332 m) (Figure 12a);
- Amalgam sample with a diameter of 9.05 mm (0.00905 m), length of 25.27 mm (0.02527 m) (Figure 12b).

These samples were weighed with the ELB300 electronic scale, as can be seen in Figure 12.

After weighing the samples, the following data were obtained:

- The composite material sample (Figure 12a) weighed 2.24 g (0.00224 kg);
- The amalgam sample (Figure 12b) weighed 18.42 g (0.01842 kg).

Applying Formulas (2) and (3), the two densities were determined:

- The density of the composite material is ρ_c_ = 1052.257 kg/$m^{3}$;
- The density of the amalgam is ρ_a_ = 11,333.9 kg/$m^{3}$.

Centralizing the data from the literature with those obtained through previous calculations, the values in Table 2 were obtained.

#### 2.7.3. Dividing Virtual Models into Finite Elements

**Figure 13:**
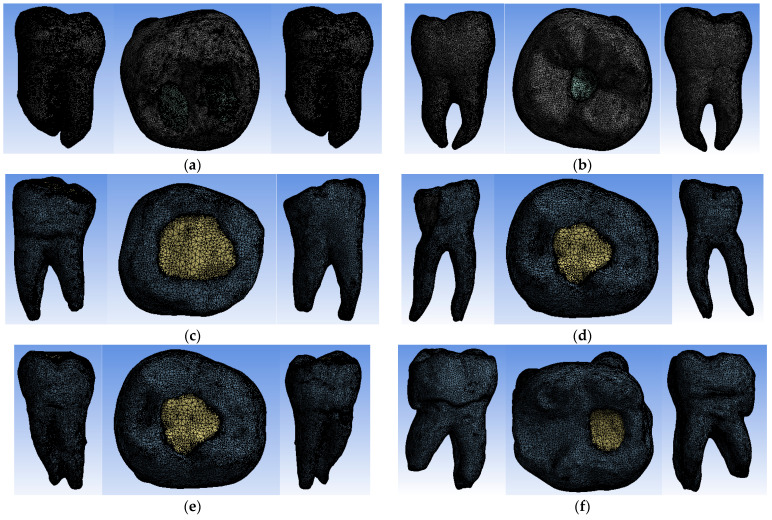
Finite element structure of virtual models (yellow color to highlight the access cavity) :(**a**) Truss Ac; (**b**) UltraAC; (**c**) TradAC; (**d**) CariesAC; (**e**) ConsAC; (**f**) RestoAC.

The virtual models were divided into finite elements. Tetrahedron-type finite elements were used. Figure 13 shows the finite element structure consisting of the following:

− 1,637,959 nodes and 1,036,362 finite elements for the virtual model of the molar with Truss AC after the coronal obturation;
− 1,076,862 nodes and 670,028 finite elements for the virtual model of the molar with UltraAC;
− 737,746 nodes and 455,890 finite elements for the virtual molar model with TradAC;
− 670,215 nodes and 421,462 finite elements for the virtual molar model with CariesAC;
− 1,231,306 nodes and 783,696 finite elements for the virtual molar model with ConsAC;
− 948,445 nodes and 595,301 elements finished for the virtual model of the molar with RestoAC.

#### 2.7.4. Imposing Mechanical Constraints in Finite Element Simulations

**Figure 14:**
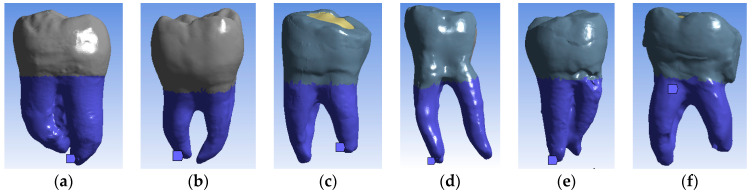
Virtual fixed surfaces of analyzed molars (highlighted with blue) (**a**) TrussAC; (**b**) UltraAC; (**c**) TradAC; (**d**) CariesAC; (**e**) ConsAC; (**f**) RestoAC.

Molars are fixed into the maxillary or mandibular bone through periodontal ligament which is a union between the roots of the teeth and the inner wall of the alveolar bone socket. To simplify the study, the surfaces of the roots were considered fixed, as shown in Figure 14 (in blue).

#### 2.7.5. Imposition of Bruxism-Specific Mechanical Force Loading System to Simulate Mechanical Constraints in Finite Element Simulations

**Figure 15:**
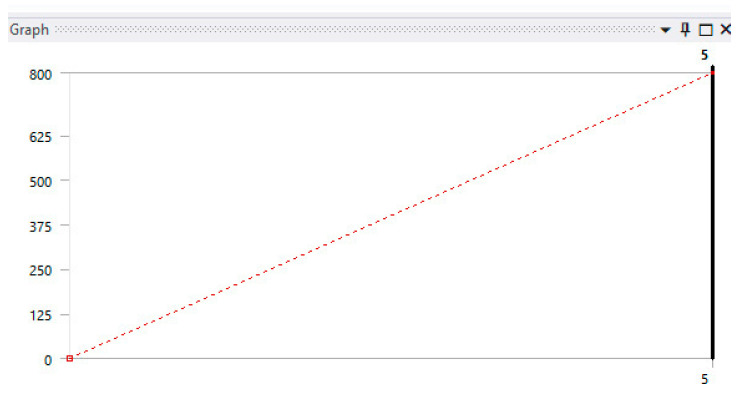
Bruxism-specific force definition chart.

Analyzing the specialized literature [39,40,41], it was found that the values of the forces that develop in bruxism are in the range of 200–800 N. To analyze such an unfavorable situation, it was considered that, in the studied case, the force should vary between the values 0–800 N, and the duration should be five seconds. Figure 15 shows the force variation graph taken from its definition in Ansys Workbench.

**Figure 16:**
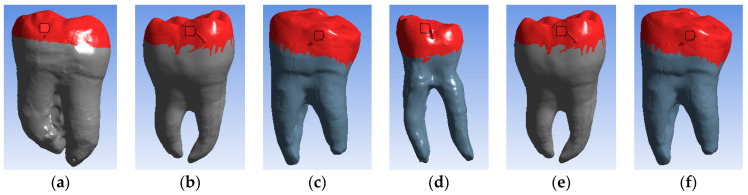
Action surfaces of forces (the forces direction is highlighted by arrows and the surface with red): (**a**) TrussAC; (**b**) UltraAC; (**c**) TradAC; (**d**) CariesAC; (**e**) ConsAC; (**f**) RestoAC.

It was considered that this force acts on the hard tissues at the top of the crowns. For the six analyzed situations, these surfaces are shown in Figure 16 (colored in red).

## 3. Results

The simulations were performed for the two coronary restoration materials, a bulk fill composite (Voco X-tra fil) and amalgam type material (Composition—Ag 69.2%, Sn 18.6%, Cu 11.9%, Zn 0.3%).

### 3.1. Numerical Results Obtained for Molars Restored with Bulk Fill Resin Composite and Bruxism-Specific Loading

**Figure 17:**
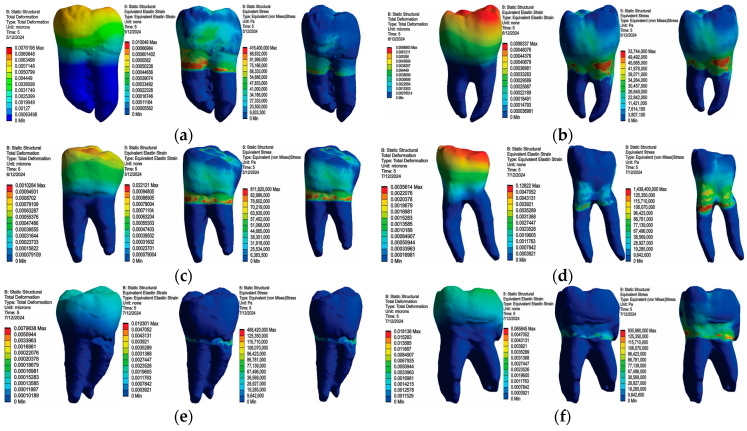
Map of displacements, strains and stress: (**a**) TrussAC; (**b**) UltraAC; (**c**) TradAC; (**d**) CariesAC; (**e**) ConsAC; (**f**) RestoAC.

In Figure 17, maps of displacements, strains and stress are shown for all access cavities used: TrussAC, UltraAC, TradAC, CariesAC, ConsAC, RestoAC. 

### 3.2. Numerical Results Obtained for Amalgam and Bruxism-Specific Loading (Figure 18)

**Figure 18:**
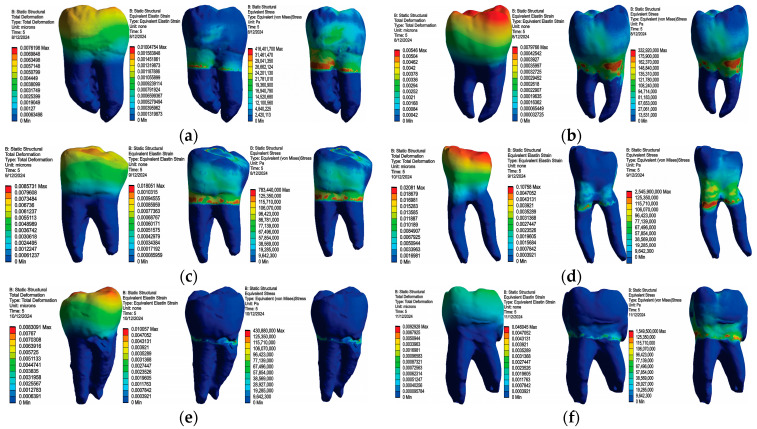
Map of displacements, strains and stress: (**a**) TrussAC; (**b**) UltraAC; (**c**) TradAC; (**d**) CariesAC; (**e**) ConsAC; (**f**) RestoAC.

In Figure 18, maps of displacements, strains and stress are shown for all access cavities used: TrussAC, UltraAC, TradAC, CariesAC, ConsAC, RestoAC.

**Figure 19:**
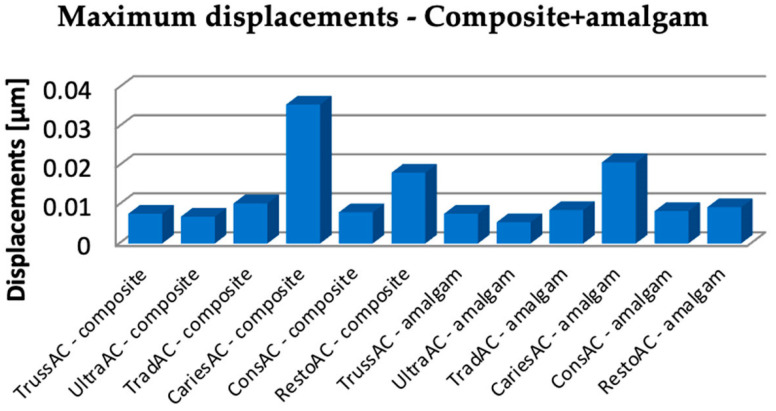
Comparison chart for maximum displacements.

**Figure 20:**
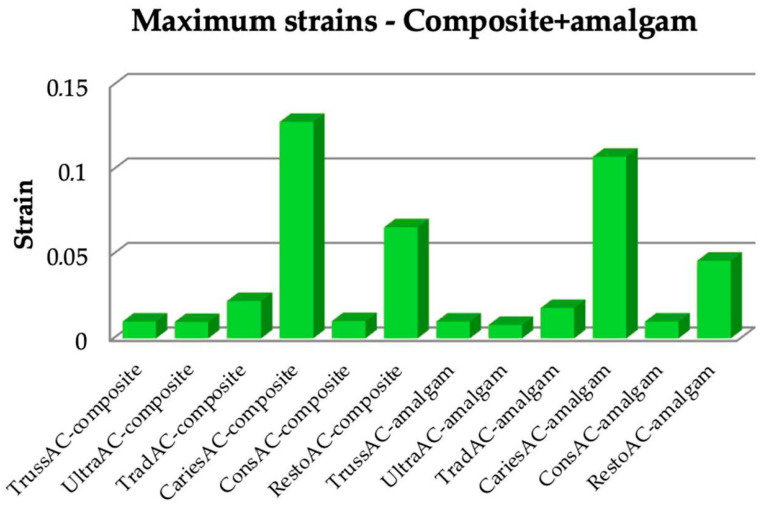
Comparison chart for maximum strains.

**Figure 21:**
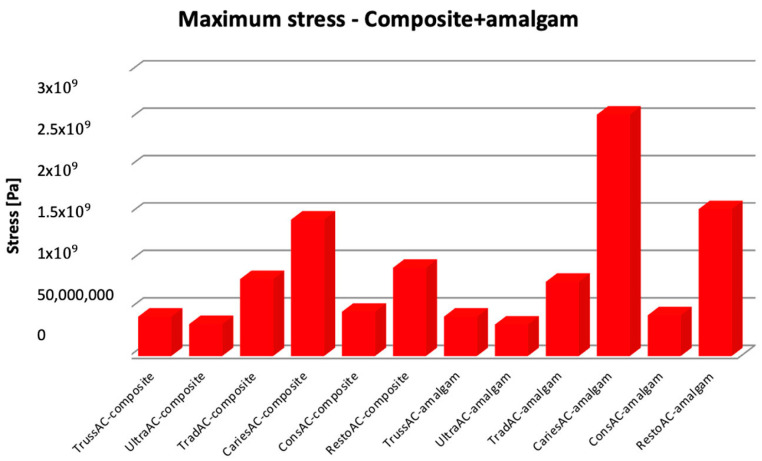
Comparison diagram for maximum stress.

The Microsoft Office package was used to organize the data extracted from the result maps. Thus, a comparative diagram was defined for maximum displacements (composite and amalgam), for strains (composite and amalgam), and for stress (composite and amalgam), the diagrams being presented in Figure 19, Figure 20 and Figure 21. 

Analyzing the obtained data, it can be seen that the restoration with amalgam registered lower displacement values in case of UltraAC, TradAC, CariesAC and RestoAC cavities. The composite restoration recorded lower displacement values in the case of ConsAC. 

Analyzing the obtained data, it can be seen that the restoration with amalgam registered lower deformation values in all types of access cavities.

Analyzing the obtained data, the restoration with amalgam recorded lower stress values in the case of UltraAC, TradAC, and ConsAC cavities. The composite restoration recorded lower displacement values in case of CariesAC and RestoAC. For TrussAC, the stress values were approximately equal for both materials.

## 4. Discussion

The evolution of teeth with endodontic treatment is a subject of great interest for specialists in the field of dentistry and is intensely debated in the specialized literature [42,43].

Fractures of endodontically treated teeth have a high prevalence [44,45], and the most important factors considered are large access cavities [46,47], excessive canal instrumentation, post space preparation [6], tissue changes due to substances used during root canal treatment [48], and large coronary restorations [45,49].

Root fractures in endodontically treated teeth have a prevalence between 3.69% and 25% [50]. Regarding coronary fractures, data from the literature are more limited. Thus, in a retrospective study from 2003, it was found that less than 7% of teeth with endodontic treatment had coronal fractures [51].

The role of the access cavity in the evolution of endodontically treated teeth is reflected in the types of cavities that have been proposed over time.

Several studies have shown that the loss of hard dental structure during the endodontic access preparation is one of the main causes for the fragility and higher risk of fracture of the endodontically treated teeth [48,52,53]. At the extremes of the types of cavities used in dental practice are traditional cavities and ninja cavities.

Traditional cavities are made to facilitate the endodontic treatment and to obtain a straight-line access to root canals. During access cavity preparation, a significant amount of tooth structure is destroyed [47]. Ninja cavities are proposed to reduce the loss of hard tooth structure [9]. They aim to preserve the hard structure of the tooth by preserving as much dentin as possible, including the pulp chamber roof, to prevent fracture during and after endodontic treatment [2,5]. In a 2014 study, Krishan et al. stated that, of endodontically treated teeth, approximately 4.6–7.5%, predominantly molars and premolars, are extracted 4–5 years after treatment [54,55]. These extractions are due to fractures in approximately 47% of cases [46,56,57]. Tamse et al. [58] and Zadik et al. [59] observed a high prevalence of fractures in endodontically treated mandibular molars, 24% and 44.6%, respectively.

Starting from the findings that fractures occur more often in endodontically treated mandibular molars than other categories of teeth, the present “in silico” study was carried out, through FEM, based on “in vitro” models of mandibular molars and highlighting the stress, strains and displacements which develop in the tissues of the mandibular molars with various forms of access cavities, following the specific loads of bruxism. The finite element method provides a versatile and powerful analytical tool that allows the examination of how stresses are transmitted in materials, overcoming certain ethical and methodological limitations and increasing the accuracy of the process [60].

The study demonstrated that the access cavity design, specifically the amount of remaining hard tissues, influenced the level of stress developed in the dental hard structures and its location. The ninja cavity revealed the lowest stress levels compared to the traditional access cavity, which revealed increased stress levels. In the specialized literature, the comparative approach of these types of access cavities is of recent date and includes clinical, “in vitro” and “in silico” studies.

Clinical studies highlight the advantages of traditional cavities for the clinical phases of endodontic treatment, without considering the prognosis of endodontically treated teeth [10,61].

Regarding the resistance of teeth depending on the access cavity design, several studies have highlighted the disadvantages of traditional cavities. Based on a review of “in vitro” studies published in 2018, Silva et al. pointed out that preservation of tooth structure, including pericervical dentin, could improve the fracture resistance of restored teeth [62].

Based on an “in vitro” study from 2017, Plotiono et al. evaluated the fracture resistance of endodontically treated teeth with different access cavity designs and found that teeth with traditional access cavities have a lower fracture resistance than those with minimally invasive cavities [63].

The results of another “in silico” FEM study from 2018 showed higher stress values in teeth with a traditional access cavity compared to those with a ninja cavity [64]. Therefore, preservation of cervical dentin may increase the fracture resistance of teeth [64].

Several studies using FEM have shown that when a greater amount of hard tissue was preserved, better fracture resistance was recorded [65,66]. As the volume of the access cavity increased, the stress concentrated in the cervical region increased [66]. In a study conducted in 2021, Jiang et al. concluded that the minimally invasive approach could reduce stress distribution in the cervical area [65].

The results obtained in the present study overlap with those in the literature. The present study showed higher stresses in the cervical region, which is a critical area for load transfer from the occlusal surface to the root.

However, contrary to these results, Saeed in 2021 conducted a review based on “in vitro” studies, from which it emerged that it cannot be established whether traditional access cavities are more advantageous than conservative cavities and additional studies are needed [67]. Mohamed Kataia conducted an “in silico” study in 2023 on two types of cavities (traditional and conservative) and concluded that reducing the size of the access cavity does not necessarily reflect positively on the biomechanical behavior of the tooth [68]. However, regarding this last study, we note that both access cavities used can be considered conservative by their size and design.

The fracture resistance of endodontically treated teeth is a topic addressed in several studies, focusing on the technique and the restorative material used [69,70,71]. The present study considered amalgam and composite restoration of molars. Amalgam has been used traditionally to restore endodontically treated posterior teeth and can be used as a definitive restoration or as an abutment reconstruction modality for a full-coverage crown. The failure rate of endodontically treated teeth and vital teeth has been shown to be the same when restored with extended amalgam restorations [72]. Several studies have claimed that it is beneficial to anchor the amalgam filling in the root canals to increase the retention of the restoration when the remaining wall thickness was less than 4 mm [73]. However, Tamse et al. compared 49 mesial roots extracted due to vertical fractures with 52 mesial mandibular roots without fractures and found that 67.3% of vertically fractured roots had amalgam retention created in the coronal part (1–2 mm) of the root [58]. The authors suggested that the removal of dentin from the coronal part of a fracture-susceptible root and condensation of amalgam in a root where the dentin layer has thinned contributes to vertical fracture development. In fact, it is the pericervical dentin which, according to the present study, represents an area of stress concentration.

Regarding the restoration of teeth with composite resins, studies suggest that composite resins provide 87% of the initial stiffness of endodontically treated teeth with an average survival period of 13.4 years [74]. It was also concluded that cavities with up to three surfaces could be successfully restored with adhesive composite resins [74,75].

The way to restore endodontically treated teeth is currently a subject of debate, going as far as recommending mixed composite-amalgam fillings [27,76].

According to the present study, the lowest level of stress displacements and strains were recorded for amalgam restorations in the case of UltraAC, TradAC and ConsAC cavities. Amalgam should therefore be considered as the first option for restoring endodontically treated teeth with these types of cavities.

To our knowledge, there are no studies in the specialized literature that address the behavior of so many cavity types and of these two restorative materials. There is only one study that studied the behavior of a single type of cavity restored with composite [28].

In 2019, Rodrigues et al. [28] conducted an FEM study in which he showed that, for endodontically treated mandibular molars with coronal destruction, the stress is localized in the coronal structures. After the coronal restoration with composite, the stress is redistributed in the root part [28]. The same stress redistribution after coronal restoration was also noted in the present study in the case of cavities with the highest loss of dental tissues (CariesAC), for both restorative materials. Comparing the stress level for the two restorative materials, it was found that the amalgam generated the highest stress level also for CariesAC.

Knowledge of the behavior of restorative materials in relation to dental tissues is particularly important for the success of restorations [77]. However, in addition to the biomechanical properties of the restorative materials, the stress to which the restored teeth will be subjected must also be considered. The teeth in the posterior region are exposed to functional and parafunctional forces with various amplitudes and directions [78]. The value of the forces generated by the masticatory muscles varies from 10 N to 431 N [79] up to 800 N as tested in this study [39,40,41].

The null hypothesis was not verified, revealing different levels of stress depending on the design of the access cavity.

The present study stands out for the fact that it studied biomechanical behavior in the case of six types of access cavities in the context of the elements of dental morphology specific to molars.

The limitations of this study are given by the fact that the FEM models of the molars did not include the entire jaw, excluding the effects of periodontal ligaments and alveolar bone so that the results represent only the response of the hard structures of the teeth. In the case of restorative materials, the cavities were filled uniformly, without voids, representing ideal situations. Another limitation of the study is given by the fact that structural changes of the analyzed teeth and the presence of possible cracks in the dental structures were not considered.

## 5. Conclusions

The study highlighted significant differences in stress distribution between the six types of access cavities. The most unfavorable access cavity for stress distribution in hard tissues was the CariesAC, which involves the removal of the largest amount of hard tissue among the studied models. The highest level of stress for all types of access cavities was recorded at the level of the pericervical dentine. Analyzing the behavior of the two restoration materials, the lowest level of displacements, strains, and stress was highlighted in the case of UltraAC, TradAC, and ConsAC cavities for amalgam. The highest level of stress was highlighted in the case of CariesAC, especially for amalgam. The results obtained in this study have practical applicability for dentists and should guide doctors towards a conservative attitude with the preservation of as much hard tissue as possible and the differentiated use of restorative materials depending on the amount of tissue lost through the creation of the access cavity.

## Figures and Tables

**Table 1:** The technical characteristics of the scanner.

Characteristics	3D Systems Capture 3D
Net weight	1.35 kg
Size (L × l × H)	276 × 74 ×49 mm
Data capture rate	98,500 points/scan
Resolution	0.110 mm la 300 mm 0.180 mm la 480 mm
Accuracy	0.060 mm
Standard distance	300 mm
Scan depth	180 mm
Field of view	124 × 120 mm (zoom in) 190 × 175 mm (zoom out)

**Table 2:** Physical and mechanical properties of materials used in FEM.

Material Name	Density [kg/m^3^]	Modulus of Elasticity E [Pa]	Poisson’s Ratio
Enamel	2.958	7.79 × 10^10^	0.3 [33]
Dentine	2.140	1.76 × 10^10^	0.25 [33]
Gutta-percha	1.000	6.1 × 10^6^	0.49 [37]
Amalgam	11.334	5 × 10^10^	0.29 [34]
Bulk fill composite	1.052	1.43 × 10^10^	0.24 [38]

## Data Availability

The authors declare that the data of this research are available from the corresponding authors upon reasonable request.
